# Essential Role of Growth Hormone and IGF-1 in Therapeutic Effect of Ghrelin in the Course of Acetic Acid-Induced Colitis

**DOI:** 10.3390/ijms18061118

**Published:** 2017-05-24

**Authors:** Piotr Ceranowicz, Zygmunt Warzecha, Jakub Cieszkowski, Dagmara Ceranowicz, Beata Kuśnierz-Cabala, Joanna Bonior, Jolanta Jaworek, Tadeusz Ambroży, Krzysztof Gil, Rafał Olszanecki, Małgorzata Pihut, Artur Dembiński

**Affiliations:** 1 Department of Physiology, Faculty of Medicine, Jagiellonian University Medical College, 31-531 Cracow, Poland; piotr.ceranowicz@uj.edu.pl (P.C.); jakub.cieszkowski@uj.edu.pl (J.C.); dagmara.ceranowicz@uj.edu.pl (D.C.); mpdembin@cyf-kr.edu.pl (A.D.); 2 Department of Pediatrics, Gastroenterology and Nutrition, University Children’s Hospital, Faculty of Medicine, Jagiellonian University Medical College, 30-663 Cracow, Poland; 3 Department of Diagnostics, Chair of Clinical Biochemistry, Faculty of Medicine Jagiellonian University Medical College, 31-501 Cracow, Poland; mbkusnie@cyf-kr.edu.pl; 4 Department of Medical Physiology Faculty of Health Sciences, Jagiellonian University Medical College, 31-126 Cracow, Poland; joanna.bonior@uj.edu.pl (J.B.); jolanta.jaworek@uj.edu.pl (J.J.); 5 Department of Theory of Sport and Kinesiology, Faculty of Physical Education, University of Physical Education, 31-571 Cracow, Poland; tadek@ambrozy.pl; 6 Department of Pathophysiology, Faculty of Medicine, Jagiellonian University Medical College, 31-121 Cracow, Poland; krzysztof.m.gil@uj.edu.pl; 7 Department of Pharmacology, Faculty of Medicine, Jagiellonian University Medical College, 31-531 Cracow, Poland; rafal.olszanecki@uj.edu.pl; 8 Department of Prosthetic Dentistry, Faculty of Medicine, Jagiellonian University Medical College, 31-155 Cracow, Poland; malgorzata.pihut@uj.edu.pl

**Keywords:** growth hormone, ghrelin, insulin-like growth factor-1, colitis, the pituitary gland

## Abstract

Previous studies have shown that ghrelin exhibits a protective and therapeutic effect in the gut. The aim of the present study was to examine whether administration of ghrelin affects the course of acetic acid-induced colitis and to determine what is the role of growth hormone (GH) and insulin-like growth factor-1 (IGF-1) in this effect. In sham-operated or hypophysectomized male Wistar rats, colitis was induced by enema with 1 mL of 3% solution of acetic acid. Saline or ghrelin (given at the dose of 8 nmol/kg/dose) was administered intraperitoneally twice a day. Seven days after colitis induction, rats were anesthetized and the severity of the colitis was assessed. Treatment with ghrelin reduced the area of colonic mucosa damage in pituitary-intact rat. This effect was associated with increase in serum levels of GH and IGF-1. Moreover, administration of ghrelin improved blood flow in colonic mucosa and mucosal cell proliferation, as well as reduced mucosal concentration of proinflammatory interleukin-1β (IL-1β) and activity of myeloperoxidase. Hypophysectomy reduced serum levels of GH and IGF-1 and increased the area of colonic damage in rats with colitis. These effects were associated with additional reduction in mucosal blood follow and DNA synthesis when compared to pituitary-intact rats. Mucosal concentration of IL-1β and mucosal activity of myeloperoxidase were maximally increased. Moreover, in hypophysectomized rats, administration of ghrelin failed to affect serum levels of GH or IGF-1, as well as the healing rate of colitis, mucosal cell proliferation, and mucosal concentration of IL-1β, or activity of myeloperoxidase. We conclude that administration of ghrelin accelerates the healing of the acetic acid-induced colitis. Therapeutic effect of ghrelin in experimental colitis is mainly mediated by the release of endogenous growth hormone and IGF-1.

## 1. Introduction

Ghrelin, a 28-amino acid peptide, was originally isolated from rat and human stomach by Kojima et al. in 1999 [1]. The stomach is the main source of endogenous ghrelin in the body [2]. Ghrelin is formed from its 117-amino acid precursor: preproghrelin [3]. The preproghrelin molecule consists of a 23-amino acid signal sequence and the 94-amino acid proghrelin, which is further processed to acyl-ghrelin, des-acyl ghrelin, and obestatin [4]. Acyl-ghrelin, which is present in serum in markedly smaller quantities than des-acyl ghrelin, was recognized to be an active form of this hormone because acylation is necessary to activate growth hormone secretagogue receptor (GHSR-1a), currently known as ghrelin receptor [5]. Before secretion of acyl-ghrelin, proghrelin undergoes posttranslatory esterification, hydroxyl group of serine-3 residue is acylated mainly by octanoic acid and, to a lesser extent by decanoic acid [6]. This modification is achieved by the ghrelin *O*-acyltransferase (GOAT), a member of the membrane-bound *O*-acyltransferase (MBOAT) family [7,8].

Des-acyl ghrelin does not activate ghrelin receptor, GHSR-1a, but several studies suggest that this form of ghrelin exhibits some GHSR-1a-independent biological activities, such as a prevention of skeletal muscle atrophy [9], protection of endothelial cells and cardiomyocytes in the heart [10], or influence on glucose metabolism [6]. These observations may indicate that the biological activity of des-acyl ghrelin is dependent on a new, yet unrecognized ghrelin receptor, different from GHSR-1a, but to date its presence has not been established. On the other hand, there are studies showing that some effects of des-acyl ghrelin such as adipogenic action in tibial marrow is dependent on des-acyl ghrelin acylation to acyl-ghrelin by GOAT and activation of GHSR-1a [11].

Ghrelin receptor is mainly present in the pituitary gland and hypothalamus, but it also occurs in lower amounts in other central and peripheral tissues [12]. Acting on its receptor in the anterior lobe of the pituitary gland, ghrelin strongly and dose-dependently stimulates secretion of growth hormone [1]. Besides the release of growth hormone, ghrelin has other biological effects, such as stimulation of gastric motility [13], appetite and fat accumulation in rats [14,15]. Stimulation of food intake has also been observed in humans [16]. Moreover, ghrelin-growth hormone axis and ghrelin *O*-acyltransferase play an essential role in growth hormone-mediated survival in fasted mice [8,17].

There are studies showing that ghrelin protects several organs, including the heart [18], kidney [19], and brain [20], against damage caused by ischemia, as well as reduces the sepsis-induced acute lung injury and mortality in rats [21]. In the gastrointestinal tract, prophylactic administration of ghrelin inhibits the development of experimental ulcers of gastric mucosa induced by ethanol [22], stress [23], and alendronate [24], as well as accelerates the healing of ulcers in the upper part of gastrointestinal tract induced by acetic acid [25], ethanol [26], or cysteamine [27]. It was also shown that ghrelin inhibits the development of different types of experimental pancreatitis [28,29] and accelerates the regeneration of the pancreas in the course of this inflammation [30,31]. In addition, ghrelin has a therapeutic effect in oral ulcers [32].

The role of ghrelin in inflammatory bowel diseases is not clear. Clinical studies have indicated that patients in the acute phase of Crohn’s disease and ulcerative colitis have higher circulating ghrelin levels than healthy individuals [33,34,35]. Moreover, the increased expression of ghrelin in the mucosa of the large intestine was found in patients with ulcerative colitis [36] and Crohn’s disease [37]. Some experimental studies have shown the therapeutic effect of ghrelin administration in trinitrobenzene sulfonic acid (TNBS)-induced colitis in rats [36] and mice [38]. On the other hand, studies conducted by De Smet et al. indicate that endogenous and exogenous ghrelin enhance the inflammatory process in the course of dextran sodium sulfate-induced colitis in mice [39].

The aim of this study was to determine the effect of ghrelin administration on the course of acetic acid-induced colitis in rats. Moreover, we examined whether ghrelin affects the course of colitis directly or whether the effects of ghrelin are mediated by its influence on growth hormone and IGF-1 secretion. For this reason, studies have been performed in pituitary-intact and hypophysectomized rats.

## 2. Results

**Figure 1:**
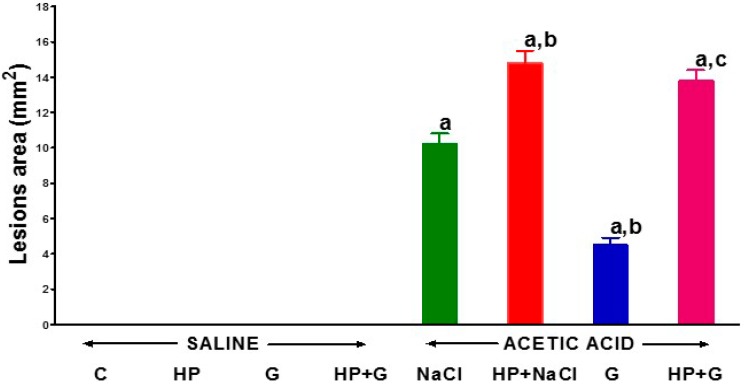
The effect of intraperitoneal administration of saline (NaCl) or octanoylated ghrelin (G) and induction of colitis (ACETIC ACID) on the area of colonic damage in pituitary-intact or hypophysectomized (HP) rats. Mean ± standard error. *n* = 10 animals in each group. ^a^ *p* < 0.05 compared to pituitary-intact saline-treated control rats without induction of colitis (C), ^b^ *p* < 0.05 compared to pituitary-intact rats treated with saline after induction of colitis (ACETIC ACID + NaCl); ^c^ *p* < 0.05 compared to pituitary-intact rats treated with ghrelin after induction of colitis (ACETIC ACID + G).

In the description of our results, we use the term ghrelin to denote active octanoylated form of ghrelin. The severity of colonic damage was assessed 7 days after enema with saline or induction of colitis by enema with acetic acid solution. Neither removal of the pituitary gland, nor intraperitoneal administration of ghrelin caused the damage to the mucosa of the colon in rats exposed to enema with saline (Figure 1). Seven days after the induction of colitis by acetic acid enema, in pituitary-intact rats treated i.p. with saline, mucosal damage in the colon reached the area of 10.2 ± 0.6 mm^2^ (Figure 1). Hypophysectomy carried out before induction of colitis led to an increase in colonic lesion area by 45%. The administration of ghrelin after induction of colitis accelerated the healing of colonic mucosa in rats with the intact pituitary gland. In those rats, 7 days after induction of colitis, the area of colonic damage was reduced by 66% in comparison to the area of lesions observed in saline-treated rats with the intact pituitary gland (Figure 1). In contrast, administration of ghrelin did not exhibit any beneficial effect on the size of the colitis-induced mucosal lesions in the colon in hypophysectomized rats (Figure 1).

**Figure 2:**
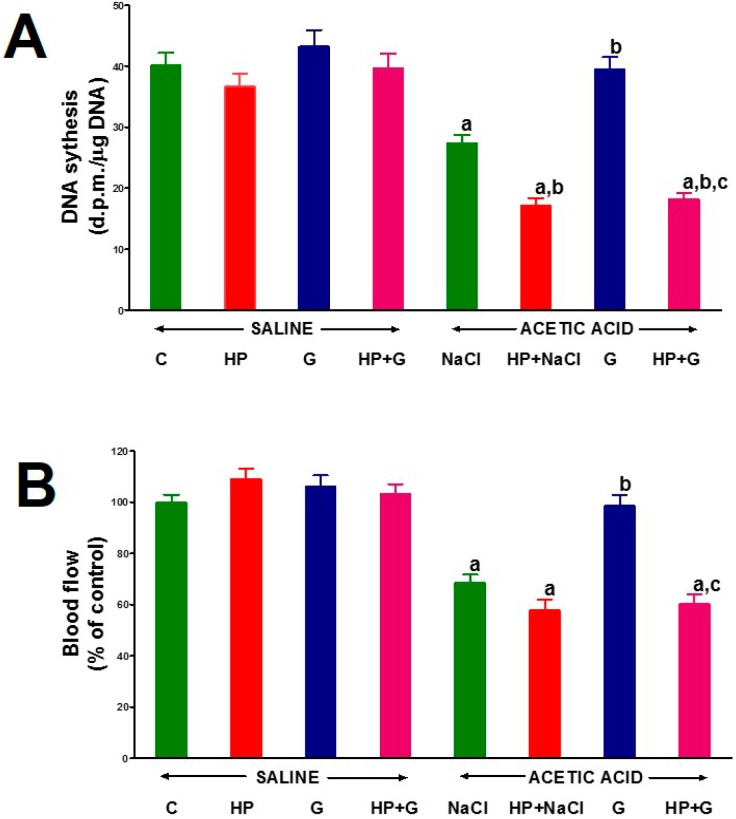
The effect of intraperitoneal administration of saline (NaCl) or octanoylated ghrelin (G) and induction of colitis (ACETIC ACID) on DNA synthesis (**A**) and blood flow (**B**) in colonic mucosa in pituitary-intact or hypophysectomized (HP) rats. Mean ± standard error. *n* = 10 animals in each group. ^a^ *p* < 0.05 compared to pituitary-intact saline-treated control rats without induction of colitis (C), ^b^ *p* < 0.05 compared to pituitary-intact rats treated with saline after induction of colitis (ACETIC ACID + NaCl); ^c^ *p* < 0.05 compared to pituitary-intact rats treated with ghrelin after induction of colitis (ACETIC ACID + G).

Neither removal of the pituitary gland, nor administration of ghrelin, nor a combination of those procedures significantly affected DNA synthesis in colonic mucosa in rats without induction of colitis (Figure 2A). Induction of colitis reduced mucosal DNA synthesis in the colon. Seven days after induction of colitis, DNA synthesis in the colonic mucosa was significantly decreased by 32% in animals with the intact pituitary gland compared to those observed in the control group without colitis (Figure 2A). Administration of ghrelin caused statistically significant and almost complete reversal of DNA synthesis drop in animals with the intact pituitary gland. Hypophysectomy performed before induction of colitis led to an additional and significant decrease in DNA synthesis in the colonic mucosa. Moreover, administration of ghrelin failed to affect DNA synthesis in the colonic mucosa in hypophysectomized rats with colitis (Figure 2A).

Hypophysectomy, as well as intraperitoneal administration of ghrelin for six days, had no significant effect on the flow of blood through the mucosa of the large intestine in rats without induction of colitis (Figure 2B). Colitis significantly reduced blood flow in colonic mucosa. Seven days after the induction of colitis, mucosal blood flow in the colon of pituitary-intact rats with colitis was reduced by 31.5% in comparison to that observed in control pituitary-intact rats without colitis. Hypophysectomy led to an additional decrease in colonic blood flow in rats with colitis, but this effect was not statistically significant (Figure 2B). The administration of ghrelin after the induction of colitis caused almost a complete reversal of the colitis-evoked decrease in blood flow through the colonic mucosa in animals with the intact pituitary gland. In hypophysectomized rats with colitis, ghrelin was without any effect on blood flow through the colonic mucosa (Figure 2B).

**Figure 3:**
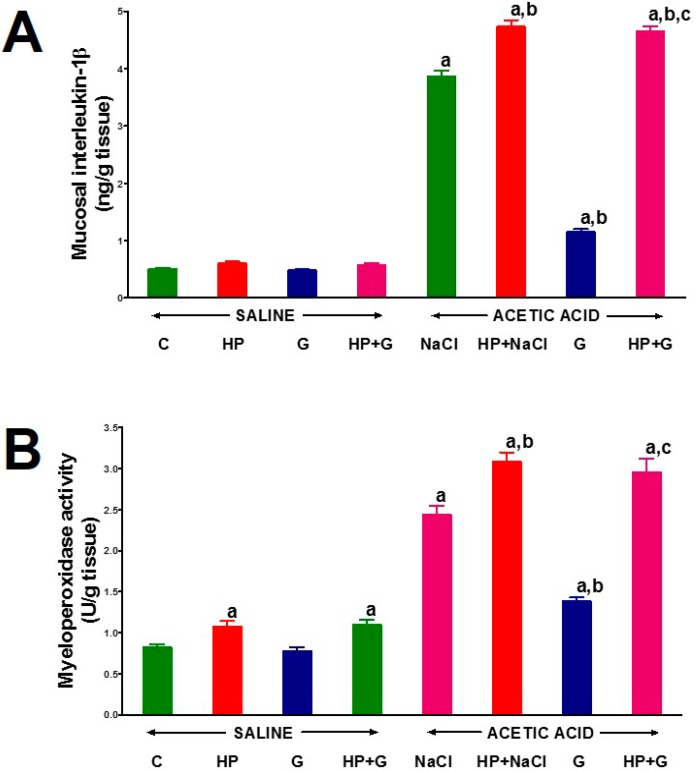
The effect of intraperitoneal administration of saline (NaCl) or octanoylated ghrelin (G) and induction of colitis (ACETIC ACID) on interleukine-1β concentration (**A**) and myeloperoxidase activity (**B**) in colonic mucosa in pituitary-intact or hypophysectomized (HP) rats. Mean ± standard error. *n* = 10 animals in each group. ^a^ *p* < 0.05 compared to pituitary-intact saline-treated control rats without induction of colitis (C), ^b^ *p* < 0.05 compared to pituitary-intact rats treated with saline after induction of colitis (ACETIC ACID + NaCl); ^c^ *p* < 0.05 compared to pituitary intact rats treated with ghrelin after induction of colitis (ACETIC ACID + G).

Hypophysectomy, administration of ghrelin for 6 days, or the combination of these two factors had no impact on the concentration of interleukin-1β in the mucosa of the large intestine in animals without induction of colitis (Figure 3A). Induction of colitis in animals with the intact pituitary gland resulted in a more than 7-fold increase in interleukin-1β concentration in colonic mucosa. In hypophysectomized rats, induction of colitis led to a more than 9-fold increase in mucosal concentration of interleukin-1β in the colon in comparison to a value observed in control pituitary-intact rats without colitis. In rats with the intact pituitary gland, treatment with ghrelin after the induction of colitis resulted in a more than 3-fold reduction in interleukin-1β concentration in the colonic mucosa. In contrast, administration of ghrelin after the induction of colitis had no effect on the levels of interleukin-1β in the mucosa of the large intestine in hypophysectomized rats (Figure 3A).

Six-day intraperitoneal administration of ghrelin did not affect myeloperoxidase activity in the colonic mucosa in pituitary-intact rats without induced colitis (Figure 3B). In animals without colitis, removal of the pituitary gland resulted in a significant 32% increase in the myeloperoxidase activity in the colonic mucosa. In these rats, administration of ghrelin was without effect on hypophysectomy-induced increase in colonic myeloperoxidase activity. Induction of colitis led to an increase in colonic activity of myeloperoxidase. In animals with the intact pituitary gland, a 3-fold increase in myeloperoxidase activity was observed in the colonic mucosa in comparison to a value observed in control pituitary-intact animals without colitis. Administration of ghrelin resulted in a statistically significant inhibition of myeloperoxidase activity in the mucosa of pituitary-intact animals with colitis. The highest myeloperoxidase activity in the mucosa of the colon was observed in hypophysectomized rats with colitis (Figure 3B). Treatment with ghrelin was without any significant effect on myeloperoxidase activity in colonic mucosa in hypophysectomized rats with colitis (Figure 3B). 

**Figure 4:**
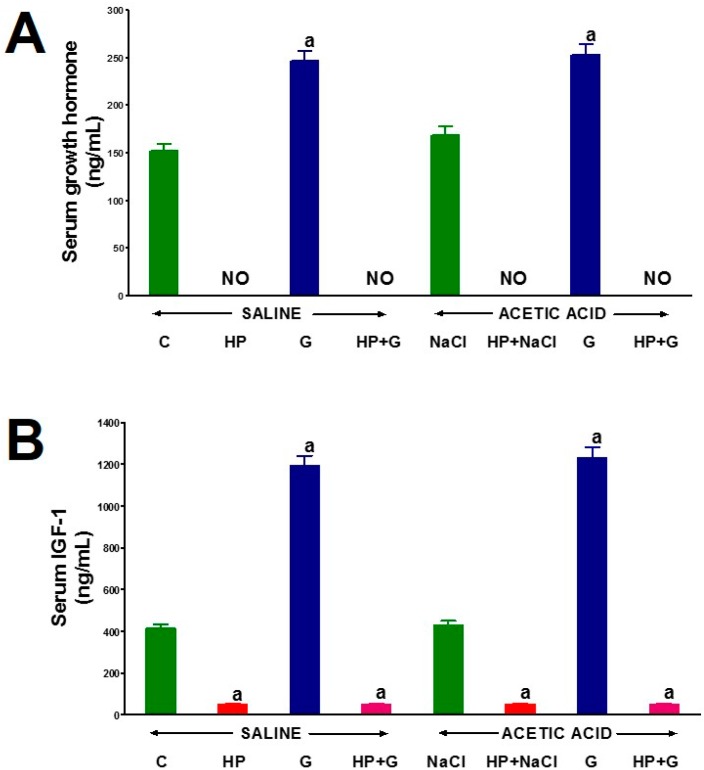
The effect of intraperitoneal administration of saline (NaCl) or octanoylated ghrelin (G) and induction of colitis (ACETIC ACID) on serum concentration of growth hormone (**A**) and IGF-1 (**B**) in pituitary-intact or hypophysectomized (HP) rats. Mean ± standard error. *n* = 10 animals in each group. ^a^ *p* < 0.05 compared to pituitary-intact saline-treated control rats without induction of colitis (C).

Serum concentration of growth hormone in animals with the intact pituitary gland and without induced colitis (control group) was 152.2 ± 7.3 ng/mL (Figure 4A). Hypophysectomy caused the elimination of serum growth hormone. Induction of colitis did not significantly affect serum growth hormone levels in pituitary-intact rat. In animals with the intact pituitary gland without colitis, a six-day administration of ghrelin resulted in a significant increase (62%) in serum growth hormone levels. A similar increase in serum growth hormone levels after administration of ghrelin was observed in pituitary-intact rats with colitis (Figure 4A).

Serum concentration of IGF-1 in control pituitary-intact animals without colitis was 415.3 ng/mL (Figure 4B). In these rats, administration of ghrelin caused a nearly 3-fold increase in serum IGF-1 levels. Hypophysectomy led to a decrease in IGF-1 levels by almost 90%. Administration of ghrelin had no effect on serum IGF-1 levels in hypophysectomized rats. The colitis by itself had no effect on serum IGF-1 levels in pituitary-intact or hypophysectomized rats in the studied period (Figure 4B).

## 3. Discussion

The present study provided some important data regarding the influence of treatment with ghrelin on the course of colitis. Our research demonstrated that ghrelin given after the development of acetic acid-induced colitis accelerates the healing of mucosal damage in the colon, as well as reduces the severity of inflammatory process in this organ. This observation is in agreement with results found in other organs of the digestive tract. A protective and/or therapeutic effect of ghrelin was identified in the oral cavity [32], stomach [25], duodenum [27], and pancreas [28,29,30,31], among others. The healing-promoting effect of ghrelin was also detected in some models of experimental colitis [36,38,40,41].

The most important finding of our present study is the observation that the therapeutic effect of ghrelin in the course of colitis is related to the release of endogenous growth hormone and IGF-1. It is well-known that ghrelin, acting on ghrelin receptor in the anterior part of the pituitary gland, stimulates a release of growth hormone [1]. Growth hormone is an endocrine regulator. It may act directly on tissues by signaling through membrane-associated growth hormone receptor (GHR), however, most growth-promoting and metabolic effects of this hormone are mediated by IGF-1 [42]. Growth hormone stimulates the release of IGF-1 in the liver and this mechanism is responsible for the delivery of the majority of circulating endogenous IGF-1. On the other hand, receptors for ghrelin, growth hormone, and IGF-1 are present in the colon and their expression is increased in pathological condition [37,43,44,45,46,47]. These data lead to the question of whether the therapeutic effect of ghrelin in the colon is mediated by this peptide directly through binding to ghrelin receptors or indirectly by release of growth hormone and IGF-1.

Our current observation confirmed that administration of ghrelin increases serum growth hormone and IGF-1 levels. In rats with the intact pituitary gland, administration of ghrelin enhanced the level of circulating growth hormone by about 60% and the level of serum IGF-1 by almost 190%. Moreover, we found that those changes in growth hormone and IGF-1 level were associated with acceleration of healing of acetic acid-induced colitis. These data strongly suggest that growth hormone and IGF-1 are involved in therapeutic effects of ghrelin in colitis. This concept is additionally supported by the observation that growth hormone and IGF-1 exhibit therapeutic effects in colitis. Christensen et al. [48] showed that treatment with growth hormone increases serum levels of IGF-1 and reduces mucosal damage score and myeloperoxidase activity in the course of trinitrobenzene sulfonic acid-induced colitis in rats. In addition, the growth hormone therapy improved body weight gain. Rats treated with growth hormone regained their initial body weight at day 7 of colitis, whereas the body weight of control rats was still below their initial body weigh [48]. Also, the increase in the release of endogenous growth hormone exhibits a therapeutic effect in experimental colitis. Using transgenic mice overexpressing growth hormone, Williams et al. tested whether enhanced plasma levels of growth hormone affect the development and the course of dextran sodium sulfate (DSS)-induced colitis [49]. They found that increased levels of endogenous growth hormone and IGF-1 do not alter the susceptibility to DSS-induced colitis but enhance survival, reduce local inflammation, and accelerate mucosal repair in this model of experimental colitis [49]. The involvement of IGF-1 in mucosal repair in mouse model of DSS-induced colitis was also reported by Chen et al. [50].

Our concept that the therapeutic effect of ghrelin in colitis is related to the release of endogenous growth hormone and IGF-1 is supported by our current data obtained from hypophysectomized rats. Hypophysectomy reduced serum concentrations of growth hormone under the detection limit, whereas serum concentrations of IGF-1 were decreased by almost 90%. These effects were associated with increased susceptibility of the colon to damage. The area of colonic lesions and severity of colitis were significantly enhanced in hypophysectomized rats in comparison to colitis observed in pituitary-intact rats.

The next form of evidence showing that the therapeutic effect of ghrelin in the course of colitis depends on the release of endogenous growth hormone and IGF-1 was obtained in hypophysectomized rats treated with ghrelin. In that group of animals, administration of exogenous ghrelin failed to affect serum levels of growth hormone or IGF-1. Moreover, the removal of the pituitary gland, the source of endogenous growth hormone, resulted in a loss of the therapeutic effect of ghrelin in this model of colitis. The area of mucosal damage and the severity of the local inflammatory process reached the same high levels as in hypophysectomized rats without treatment with ghrelin.

It must be pointed out that hypophysectomy not only removes the source of growth hormone, but also other pituitary hormones, which could affect the healing of colitis. Among them are inter alia adrenocorticotropic hormone (ACTH), prolactin, and thyroid-stimulating hormone (TSH). Moreover, previous studies showed that ghrelin significantly stimulates the release of prolactin and ACTH [51,52,53]. This last hormone, ACTH and glucocorticoids, whose synthesis and release is stimulated by ACTH, exhibit anti-inflammatory and therapeutic effects in colitis. ACTH was even used in clinical studies. A prospective, randomized, double-blind clinical trial showed that therapy with ACTH is more effective than treatment with hydrocortisone in patients with severe ulcerative colitis, who have not been previously treated with corticosteroids [54]. In addition, colitis was shown to increase plasma levels of corticosterone [55]. The above data may suggest that the deleterious effect of hypophysectomy on the course of colitis and the therapeutic effect of ghrelin in this disease in pituitary-intact rats could be independent of the release of endogenous growth hormone and IGF-1, but related to other hormones released from the pituitary gland. Fortunately, we previously performed studies on the healing effect of ghrelin in the course of acetic acid-induced gastric and duodenal ulcers [25]. As in current studies in the colon, we found that administration of ghrelin exhibits therapeutic effects in gastric and duodenal ulcers in pituitary-intact rats, whereas hypophysectomy aggravated lesions in the stomach and duodenum, and abolished the therapeutic effect of ghrelin in those ulcers. On the other hand, the use of exogenous IGF-1, at doses causing an increase in serum levels of this peptide similar to that obtained after administration of ghrelin in pituitary-intact rats, resulted in a therapeutic effect in hypophysectomized rats. This effect was similar to that observed in pituitary-intact rats treated with ghrelin. By analogy, we can expect the similar healing-promoting effect of IGF-1 in hypophysectomized rats with colitis. The above data strongly suggest that the therapeutic effect of ghrelin in the gastrointestinal tract, including the colon, is mainly related to the release of endogenous growth hormone and IGF-1.

Our present study showed that apart from the acceleration of mucosal damage healing, other mechanisms of therapeutic effect of ghrelin in colitis are also associated with the release of endogenous growth hormone and IGF-1. The therapeutic effect of ghrelin was associated with an improvement of DNA synthesis in the colonic mucosa. Epithelial cell proliferation in the gastrointestinal mucosa exhibits high dynamics and, in case of the colon, the time of cell renewal of the mucous membrane is between 3–8 days. Increased cell proliferation may cause hyperplasia of the mucosa, but it also leads to increased protection of gastrointestinal mucosa against damaging agents, as well as accelerates the restoration of mucosa integrity after damage [26,56,57]. DNA synthesis occurs in the S phase of the cell cycle, which is a preparatory phase for cell division [58]. Therefore, the rate of DNA synthesis reflects the cell vitality and cell proliferation. Administration of ghrelin failed to affect DNA synthesis in colonic mucosa in rats without induction of colitis. This observation indicates that administration of exogenous ghrelin, at the dosage used, does not lead to overstimulation of DNA synthesis and hyperplasia in the colon in rats with normal colonic mucosa. Induction of colitis in pituitary-intact rats led to a decrease in DNA synthesis below the values observed in the control animals, without colitis. In these rats, treatment with ghrelin significantly reversed the colitis-induced reduction in mucosal DNA synthesis. On the other hand, treatment with ghrelin was without any effect on mucosal DNA synthesis in the colon of hypophysectomized rats. These findings indicate that the ghrelin-induced stimulation of DNA synthesis and growth-promoting effect are related to the release of endogenous growth hormone and IGF-1.

Another interesting observation of our current study was finding that endogenous growth hormone and IGF-1 are involved in the ghrelin-induced reduction in local inflammation. The severity of local inflammation was determined by measuring the interleukin-1β concentration and activity of myeloperoxidase in colonic mucosa. Interleukin-1β plays a crucial role in the development of local inflammation and systemic acute phase response. Interleukin-1β acts directly, as well as stimulates the release of other pro-inflammatory factors, mainly interleukin-6, tumor necrosis factor-α (TNF-α) and prostaglandins [59,60]. Administration of interleukin-1 receptor antagonist was shown to prevent the rise in serum interleukin-6 and TNF-α, leading to a decrease in the severity of systemic inflammation [61]. Myeloperoxidase is an enzyme mainly expressed in neutrophils and for this reason the local activity of myeloperoxidase reflects the degree of tissue infiltration by these leukocytes [62,63]. Our current study showed that induction of colitis by acetic acid enema is associated with an increase in mucosal interleukin-1β concentration and activity of myeloperoxidase in the colon. In pituitary-intact rats, treatment with ghrelin reduced the colitis-evoked increase in mucosal concentration of those indices of inflammation. This anti-inflammatory effect of ghrelin could be a result of its direct action on ghrelin receptors present on the membranes of the immune system cells [64,65]. However, experiments performed on hypophysectomized rats indicated that the anti-inflammatory effect of ghrelin in acetic acid-induced colitis is related to the release of endogenous growth hormone and IGF-1. In hypophysectomized rats, administration of ghrelin failed to affect the colitis-evoked increase in mucosal levels of interleukin-1β and myeloperoxidase.

Adequate blood flow plays an essential role in the protection and healing of mucosa in the gastrointestinal tract [66]. Previous studies have shown that exposure of gastric mucosa to a potentially damaging agent results in little or no damage as long as suitable blood flow is maintained. On the other hand, reduction in mucosal blood flow leads to severe mucosal injury [67]. Our present study showed that induction of colitis reduces mucosal blood flow in the colon, and this effect is more pronounced in hypophysectomized rats than in pituitary-intact rats. In pituitary-intact rats, treatment with ghrelin significantly reversed the colitis-evoked reduction in colonic blood flow. In contrast, administration of ghrelin was without any effect on mucosal blood flow in the colon in hypophysectomized rats. These observations indicate that also the ghrelin-evoked improvement of blood flow in colonic mucosa in pituitary-intact rats with colitis is dependent on the release of endogenous growth hormone and IGF-1.

In contrast to our results, Gonzalez-Rey et al. [38] reported that the therapeutic effect of ghrelin in colitis is independent of the release of endogenous growth hormone and IGF-1. They used two mouse models of experimental colitis, trinitrobenzene sulfonic acid (TNBS)-induced colitis and DSS-induced colitis. In both those models of colitis, Gonzalez-Rey et al. observed that administration of exogenous ghrelin inhibited the development of colitis and accelerated recovery from this inflammation. Those results are in agreement with our findings on the role of ghrelin in experimental colitis. In the second part of their study, Gonzalez-Rey et al. administered intravenously monoclonal antibodies against growth hormone or IGF-1 or the combination of antibodies against growth hormone and IGF-1. They found that administration of those antibodies failed to affect the beneficial effect of ghrelin in the course of colitis and for this reason the authors of this paper concluded that the therapeutic influence of ghrelin in colitis is not related to the release of endogenous growth hormone and IGF-1. However, it must be noted that there are some points from the aforementioned article that make this paper difficult for univocal interpretation. First of all, Gonzalez-Rey et al. showed that the peak level of growth hormone and IGF-1 is observed about 30 min after ghrelin administration with a subsequent decrease in serum concentrations of those anabolic factors. Twenty-four hours after ghrelin administration, the serum levels of growth hormone and IGF-1 reached the baseline [38]. However, despite those observations, the authors of the paper did not use antibodies against growth hormone and IGF-1 before giving ghrelin or eventually at the same time as ghrelin. They used antibodies 2 h after ghrelin administration, resulting in at least 2-h tissue exposure to elevated levels of growth hormone and IGF-1. Moreover, it was not tested whether those antibodies were effective in lowering endogenous levels of growth hormone and IGF-1. Therefore, it is impossible to ascertain whether the therapeutic effect of ghrelin after application of antibodies against growth hormone and IGF-1 is independent of the above hormonal factors or is the result of insufficient elimination of growth hormone and IGF-1 from the body.

## 4. Materials and Methods

In the present study, we used 80 male Wistar rats weighing 180–250 g. The experimental protocol was approved by the First Local Commission of Ethics for the Care and Use of Laboratory Animals in Cracow (Permit No 20/2008 released on 21 February 2008 and Permit No 100/2007 released on 13 December 2007 with changes released 6 July 2011). During experiments, the animals were kept in cages placed in a room temperature with 12 h light-darkness cycle. Rats were randomly divided into eight groups: (1) control pituitary-intact rats treated intraperitoneally (i.p.) with saline without induction of colitis; (2) hypophysectomized rats treated i.p. with saline without induction of colitis; (3) pituitary-intact rats treated i.p. with ghrelin without induction of colitis; (4) hypophysectomized rats treated i.p. with ghrelin without induction of colitis; (5) pituitary-intact rats treated i.p. with saline after induction of colitis; (6) hypophysectomized rats treated i.p. with saline after induction of colitis; (7) pituitary-intact rats treated i.p. with ghrelin after induction of colitis; (8) hypophysectomized rats treated i.p. with ghrelin after induction of colitis.

Rats were anesthetized with ketamine (50 mg/kg, i.p., Bioketan, Vetoquinol Biowet, Gorzów Wielkopolski, Poland) and sham-operated (pituitary-intact rats) or hypophysectomized via the transauricular approach according a method described previously [68]. Two weeks after sham-operation or removal of the pituitary gland, the animals were anesthetized again with ketamine. In half of them (group 5 to 8), colitis was induced by an enema with 1 mL of 3% acetic acid solution, as described previously [69]. Groups of the animals without induction of colitis received saline administered rectally. Twenty-four hours after the induction of colitis or rectal administration of saline, animals were treated over 6 days with ghrelin or saline administered i.p. Rat recombinant octanoylated ghrelin (Yanaihara Institute, Shizuoka, Japan) was given at the dose of 8 nmol/kg according to previous studies that demonstrated that this dose of ghrelin shows a clear and reproducible protective effect in the colon [40]. The severity of colonic damage was evaluated after 7 days following rectal administration of acetic acid solution or saline. Each experimental group consisted of 10 animals.

At the end of experiments, animals were treated i.p. with saline or ghrelin and anesthetized again with ketamine. After opening the abdominal cavity and exposure of the colon, colonic blood flow was measured using a laser Doppler flowmeter (PeriFlux 4001 Master Monitor, Perimed AB, Jarfalla, Sweden), as described previously in detail [70]. Data were presented as the percentage of the value obtained in control pituitary-intact rats treated with saline without induction of colitis.

After determination of colonic blood flow, about 30 min after i.p. treatment with saline or ghrelin, blood samples were taken from the abdominal aorta and serum collected and frozen at −60 °C. Serum growth hormone concentration was determined by radioimmunoassay, using a commercial Rat Growth Hormone RIA Kit (LINCO Research, St. Charles, MO, USA). Serum IGF-1 concentration was measured by radioimmunoassay, using a commercial Mouse/Rat IGF-I RIA Kit (Diagnostic System Laboratories, Inc., Webster, TX, USA).

Then, the whole large bowel was cut out from the body, opened along its long axis, and washed with ice-cold saline. The area of colonic mucosa damage was measured using the computerized planimeter (Morphomat, Carl Zeiss, Berlin, Germany), as described previously [27,69].

Samples of colonic mucosa were collected for determination of the rate of DNA synthesis, interleukin-1β concentration, and myeloperoxidase activity. DNA synthesis in the colonic mucosa was determined by radioisotope method by measuring the incorporation of tritium-labeled thymidine ([6-3H] thymidine, 20–30 Ci/mmol; Institute for Research, Production and Application of Radioisotopes, Prague, Czech Republic) into DNA, as previously described [70]. Rate of DNA synthesis was expressed as disintegration of [^3^H]thymidine per minute per microgram of DNA (dpm/μg DNA).

Samples of the colonic mucosa for determination of interleukin-1β concentration were homogenized in phosphate buffer at 4 °C. Then homogenate was centrifuged and the concentration of interleukin-1β in the supernatant was determined using the kit BioSource Cytoscreen rat IL-1β kit (BioSource International, Camarillo, CA, USA). The concentration of interleukin-1β of the colonic mucosa was expressed in nanograms per gram of tissue.

Determination of mucosal myeloperoxidase activity was performed using the method previously described by Bradley et al. [71]. The results were expressed as a percentage of the value observed in control, pituitary-intact, rats treated with saline without induction of colitis.

Statistical analysis was performed by analysis of variance and Tukey’s test using the GraphPadPrism program (GraphPad Software, San Diego, CA, USA). Differences were considered statistically significant when *p* was less than 0.05. Results were presented as mean value ± standard error.

## 5. Conclusions

In conclusion, in colitis induced by acetic acid, ghrelin administration after the induction of inflammation exhibits a therapeutic effect in pituitary-intact rats. In contrast, hypophysectomy completely abolished the therapeutic effect of ghrelin in colitis induced by rectal administration of acetic acid. These findings suggest that the therapeutic effect of ghrelin in colitis is mainly mediated by endogenous growth hormone and IGF-1.
